# Host shutoff activity of VHS and SOX-like proteins: role in viral survival and immune evasion

**DOI:** 10.1186/s12985-020-01336-8

**Published:** 2020-05-19

**Authors:** Tianqiong He, Mingshu Wang, Anchun Cheng, Qiao Yang, Ying Wu, Renyong Jia, Mafeng Liu, Dekang Zhu, Shun Chen, Shaqiu Zhang, Xin-Xin Zhao, Juan Huang, Di Sun, Sai Mao, Xuming Ou, Yin Wang, Zhiwen Xu, Zhengli Chen, Lin Zhu, Qihui Luo, Yunya Liu, Yanling Yu, Ling Zhang, Bin Tian, Leichang Pan, Mujeeb Ur Rehman, Xiaoyue Chen

**Affiliations:** 1 Institute of Preventive Veterinary Medicine, Sichuan Agricultural University, Wenjiang, Chengdu City, Sichuan 611130 People’s Republic of China; 2 Key Laboratory of Animal Disease and Human Health of Sichuan Province, Sichuan Agricultural University, Wenjiang, Chengdu City, Sichuan 611130 People’s Republic of China; 3 Avian Disease Research Center, College of Veterinary Medicine, Sichuan Agricultural University, Wenjiang, Chengdu City, Sichuan 611130 People’s Republic of China

**Keywords:** Herpesvirus, Host shutoff, VHS, ICP27, SOX, mRNA, Immune evasion

## Abstract

**Background:**

Host shutoff refers to the widespread downregulation of host gene expression and has emerged as a key process that facilitates the reallocation of cellular resources for viral replication and evasion of host antiviral immune responses.

**Main body:**

The *Herpesviridae* family uses a number of proteins that are responsible for host shutoff by directly targeting messenger RNA (mRNA), including virion host shutoff (VHS) protein and the immediate-early regulatory protein ICP27 of herpes simplex virus types 1 (HSV-1) and the SOX (shutoff and exonuclease) protein and its homologs in *Gammaherpesvirinae* subfamilies, although these proteins are not homologous. In this review, we highlight evidence that host shutoff is promoted by the VHS, ICP27 and SOX-like proteins and that they also contribute to immune evasion.

**Conclusions:**

Further studies regarding the host shutoff proteins will not only contribute to provide new insights into the viral replication, expression and host immune evasion process, but also provide new molecular targets for the development of antiviral drugs and therapies.

## Background

The *Herpesviridae* family comprises over 100 viruses that can infect a wide variety of species of at least two animal phyla, the Chordata (mammals, birds, fishes, reptiles, and amphibians) and the Mollusca (oysters), with each member consisting of an enveloped icosahedral capsid containing a proteinaceous tegument and a dsDNA genome [1]. The *Herpesviridae* family is divided into three subfamilies (*Alphaherpesvirinae*, *Betaherpesvirinae*, and *Gammaherpesvirinae*) based on their biological properties and genome sequences [2]. The *Alphaherpesvirinae* subfamily contains HSV-1/2; the *Betaherpesvirinae* subfamily contains human cytomegalovirus (HCMV) and human herpesvirus-6A and 6B (HHV-6A and -6B) [3]; and the *Gammaherpesvirinae* subfamily contains Kaposi’s sarcoma-associated herpesvirus (KSHV), Epstein-Barr virus (EBV) and murine gammaherpesvirus 68 (MHV68) [4]. A characteristic feature that is common to all herpesvirus infections is the establishment of latent infections, a state from which the virus can be reactivated and result in recurring disease [2].

Host shutoff, on the one hand, means directly reducing the levels of cellular mRNAs or preventing their association with ribosomes and translation initiation factors that can facilitate the translation of viral mRNAs [5]. A common theme is that different viruses encode a few proteins that block host gene expression by promoting global mRNA degradation, such as the virion host shutoff (VHS) protein of HSV-1/2 [6], SOX and its homologs in gammaherpesviruses [7, 8], Nsp1 of SARS-coronaviruses [9] and PA-X from influenza A virus [10]. In cells, each protein targets host RNA polymerase II (Pol II) transcripts for cleavage and requires host Xrn1 to complete RNA degradation, although the mechanism of targeting and the position of the primary cleavage differs [11, 12]. On the other hand, HSV-1 ICP27 interacts with splicing proteins and inhibits cellular pre-mRNA splicing early after infection, resulting in a decrease in the splicing of products into cellular translation machinery; therefore, HSV-1 ICP27 also contributes to the shutoff of host protein synthesis [13, 14]. However, betaherpesviruses, such as HCMV, do not shut off host macromolecular synthesis [15]. In this review, we will discuss host shutoff mechanisms of HSV-1 and members of the *Gammaherpesvirinae* subfamilies and their roles in immune evasion.

## Main text

### mRNA processing

HSV infection leads to suppression of cellular protein synthesis through at least two distinct inhibitory pathways. In the first pathway, delivery of the VHS protein, encoded by the HSV UL41 gene, into the cytoplasm after fusion of the viral envelope with the host cell membrane. This event leads to an increase in the global mRNA degradation rate in the cytoplasm, and the precipitous decrease in the levels of most host mRNAs curtails the synthesis of the corresponding proteins [16]. VHS and its homologs are only present in the genomes of *Alphaherpesvirinae* subfamily members, and as an endoribonuclease with similar substrate specificity to RNase A, VHS triggers the rapid shutoff of host cell protein synthesis and disrupts preexisting polyribosomes [17]. The pseudorabies virus (PRV) UL41 gene-encoding protein is the homologue of the HSV UL41 protein and has a similar shutoff function [18]. In contrast, the varicella-zoster virus (VZV) open reading frame 17 (ORF17) protein, a homolog of HSV UL41 protein, can also induce RNA cleavage, but to a substantially lesser extent than HSV-1 VHS and has no major function in the VZV-mediated delayed host shutoff [19]. Interestingly, ORF17 protein is crucial for VZV replication at 37 °C [20]. Thus, VHS likely plays a fundamental and conserved role in the biology of infections caused by alphaherpesviruses, but its effect in different viral infections is distinct. In addition, VHS reduces dsRNA levels by reducing the potential for generating dsRNA and directly removing dsRNA after its formation [21]. This novel function would be important in immune evasion and may exist in other alphaherpesviruses.

Second, HSV-1 ICP27 has role in pre-mRNA polyadenylation and splicing that inhibits host mRNA maturation [22]. Eukaryotic pre-mRNAs are processed after synthesis in the nucleus and then translated in the cytoplasm, although an unusual feature of HSV-1 transcripts is that the majority are intronless (except ICP22, ICP0, ICP47, UL15, LAT and gC) and thus do not interact with the splicing machinery [23]. ICP27 interacts with and recruits cytoplasmic kinase SR protein kinase 1 (SRPK1) to the nucleus to inhibit host cell splicing, after which the unspliced host mRNAs in the nucleus cannot be exported to the cytoplasm for translation, leading to host protein synthesis shutoff [24]. Recently, ICP27 has been shown to inhibit the splicing of specific introns and promote the use of alternative 5′ splice sites (ss). Furthermore, ICP27 targets high GC content and cytosine-rich sequences that are similar to those of HSV genes spared by the VHS, possibly promoting virus-induced host shutoff [25]. In addition, transcription termination affects mRNA production and translation, and HSV-1 induces the disruption of transcription termination of host genes [26]. HSV-1 ICP27 was recently shown to block the transcription termination of host genes by inhibiting mRNA 3′ processing. Furthermore, ICP27 can act as a sequence-dependent activator of mRNA 3′ processing to promote efficient transcription termination of viral transcripts, indicating that HSV-1 ICP27 plays an important role in host shutoff [27].

Similar to alphaherpesviruses, gammaherpesviruses promote host shutoff by inducing widespread cellular mRNA degradation during the early lytic phase of viral infection [7, 8, 28]. The KSHV host shutoff RNase is not homologous to VHS, but is the alkaline exonuclease ORF37 protein, also known as SOX, a member of the PD(D/E) XK restriction endonuclease superfamily. While its homologs in other gammaherpesviruses are also host shutoff factors, SOX homologs are also present in other herpesviruses, including those such as HCMV that fail to inhibit host gene expression [11]. However, the SOX protein and its homologs (muSOX and BGLF5) in gammaherpesviruses possess both exonucleolytic DNase and RNase activities. These activities are genetically separable, and the shutoff activity does not require DNase activity, although the processing of DNA and RNA substrates requires the same catalytic center [29].

In contrast to VHS, SOX is not packaged in virion particles and is expressed with early kinetics [8]. SOX targets a degenerate motif to degrade many mRNAs in the cytoplasm [29, 30]. In addition, it induces nascent cellular mRNAs to undergo poly(A) tail extension (hyperadenylation), which prevents the export of nascent nuclear messages [31, 32]. muSOX continues to accumulate during the late stages of the viral replicative cycle and broadly targets viral mRNAs from all three kinetic classes, which generally results in a decrease in relevant viral protein levels at each class [33]. Selective inactivation of the mRNA degradation activity of muSOX results in altered protein composition of progeny virions, which ultimately impacts subsequent rounds of infection by favoring lytic cycle entry over latency [33, 34]. The deletion of BGLF5 results in the accumulation of several viral proteins during EBV infection and causes nuclear egress defects [35]. In addition, SOX and its homologs possess intrinsic RNase activity, but they cannot solely account for host shutoff in vitro [36].

Although a wide variety of mRNAs are degraded by viral endonucleases, some mRNAs contain a SOX resistance element (SRE) in their 3′ untranslated region (UTR) that prevents their degradation by multiple viral endonucleases, such as C19ORF66, IL-6 and DNA damage-inducible gene 45 (GADD45β) [37–39]. A number of ribonucleoprotein complex proteins are involved in this process, for example, nucleolin (NCL) binds the IL-6 mRNA 3’UTR and eIF4H to protect IL-6 mRNA from degradation [37], although a detailed mechanism how SREs promote the escape of mRNAs from viral endonucleases mediated decay remains unknown. Apoptosis enhancing nuclease (AEN) mRNA is also spared from SOX-mediated decay without a clear protective element in its sequence [40], and VHS cannot degrade tristetraprolin (TTP) [41]. These results suggest that multiple mechanisms can apparently promote mRNA escape. However, with the exception of the SREs, whether some mRNAs involved in the viral or cellular life cycle are spared viral endonuclease-mediated decay remains unknown, and these spared mRNAs may be needed for viral gene expression or to activate the immune response to inhibit viral replication.

### Downregulation of host mRNA translation

In eukaryotes, a key factor in translation control is eukaryotic translation initiation factor 2 (eIF2). The α subunit of eIF2 is phosphorylated by a number of kinases, including protein kinase (PKR), PKR-like endoplasmic reticulum kinase (PERK), general control nonderepressible-2 kinase (GCN2), and heme-regulated eIF2a kinase (HRI), resulting in translation arrest and, ultimately, a general translational shutoff [42]. This effect is harmful to viruses that need the host translation machinery to synthesize viral proteins. However, VHS blocks PKR activation via its endoribonuclease activity during the immediate onset of viral infection to counteract the activation of eIF2 by kinases, and VHS-defective viruses induce the phosphorylation of eIF2α [43, 44]. In addition, HSV-1 ICP27 inhibits PKR binding to dsRNA and its autophosphorylation but has no direct effect on eIF2α phosphorylation, potentially by only causing conformational changes in PKR [45].

In vitro-translated VHS exhibits endonuclease activity with no selectivity. Nevertheless, VHS shows a strong preference for mRNAs in vivo [46], degrading the 5′ end of mRNAs prior to the 3′ end, and is targeted to regions of translation initiation through its interaction with eIF4H [47]. Interestingly, several VHS mutations that abrogate its ability to bind eIF4H also abolish its mRNA-degrading activity, even though the mutant proteins retain endonuclease activity. Interestingly, several point mutations that abolish its mRNA-degrading activity also abrogate its ability to bind eIF4H, the depletion of which impedes VHS-mediated degradation [48]. Furthermore, eIF4H switches from cytoplasmic to nuclear localization during the initial shutdown of translation after viral infection [49]. However, the interaction between VHS and eIF4B or eIF4F is not sufficient to induce mRNA decay [50]. Alternatively, the targeting of VHS may depend upon its ability to interact with translation factors, whether the preferred cleavage sites are in regions of translation initiation or not [51]. However, VHS cleaves mRNAs close to AU-rich elements (AREs) in their 3′ UTRs by interacting with tristetraprolin (TTP) [52]. The internal ribosome entry site (IRES) derived from encephalomyocarditis virus (EMCV) or poliovirus acts to strongly target VHS-dependent RNA cleavage events to a narrow zone located immediately 3′ to the IRES [53]. These two degradation models require neither ribosome scanning nor interaction with translation initiation factors to select the cleavage sites. Unlike VHS, SOX has no interaction with eIF4H and cosediments with 40S ribosomal subunits, depletes polysomes, and specifically recognizes mRNAs at an early stage of translation, although the factor(s) involved SOX recruitment to its mRNA targets remain unknown [32]. During MHV68 infection, because the translation factors are unlimited, the targeting of viral mRNAs during gammaherpesvirus infection is not a mechanism to redirect the translation machinery towards host genes [34].

Cytoplasmic poly (A)-binding protein (PABPC) is a predominantly cytoplasmic protein that is required for efficient translation initiation and binds to mRNA poly(A) tails to enhance mRNA stability, translation efficiency, and quality control in the cytoplasm, in part through its interactions with the eIF4G translation initiation factor [54]. During lytic HSV-1 infection, VHS, ICP27 and other viral proteins induce the translocation of PABPC from the cytoplasm to the nucleus [55–57]. In addition, ICP27 associates with PABP and eIF4G to promote translation initiation [58]. SOX and its homologs also relocalize PABPC into the nucleus [30, 57, 59, 60], where intranuclear PABPC accumulation leads to excessive nuclear mRNAs and a block in the nuclear export of mRNAs, resulting in restricted protein expression [57].

### Boosting the expression of viral proteins

VHS directly or indirectly enhances the translation of viral mRNAs. VHS boosts the translation of viral true late mRNAs in a cell type-dependent manner and then determines the viral growth phenotype in the respective cell type, such as Hela cells [61, 62]. First, VHS refines the transition between the successive expression of viral IE, E, and late (L) genes to facilitate the turnover of all kinetic classes of viral mRNAs [63], preventing “mRNA overload” during the late stages of infection by eliminating host mRNAs and promoting the decay of viral IE and E transcripts [64]. In the absence of VHS, the half-lives of all classes of viral transcripts are dramatically increased, and the resulting accumulation of viral mRNAs overwhelms the capacity of the host translational machinery, leading to functional deficiency of the L mRNAs that are made during infection. Second, recent reports have shown that the translational defect observed for L mRNAs in the absence of VHS does not stem from one or more structural features of the affected mRNAs, since these transcripts accumulate late during infection [64]. Third, VHS is more sensitive to unspliced mRNAs than spliced mRNAs, and exon junction complexes (EJCs) may transiently protect spliced mRNAs from VHS degradation, causing a modest stimulation in translation and accumulation of spliced mRNA [65]. Thus, VHS may also enhance the expression of these viral genes depending on other functions.

To avoid multiple viral mRNAs being degraded by VHS at later times of infection in an unrestrained fashion, the viral proteins ICP27, VP13/14, VP16 or VP22 interact with and attenuate VHS RNase activity [66, 67]. Furthermore, the VP16-VP22 complex rescues the nuclear retention of VHS mRNA and the VHS-induced nuclear retention of late transcripts during HSV-1 infection, allowing for their efficient translation [67, 68]. VP13/14 stabilizes host and viral IE mRNAs and effectively blocks the degradation of E and L mRNAs, but it has no effect on the processing of AU-rich mRNAs [66, 69]. ICP27 also interacts with VHS, which may impact the stability of ARE-containing mRNAs, although this function remains controversial [70]. ICP27 and VP13/14 as nucleocytoplasmic shuttling proteins that can bind and transport RNA [71, 72], we speculate there are unknown associations between VHS, mRNA, VP13/14 and ICP27. And these interactions may also facilitate incorporation of VHS into the tegument of progeny virions. Unlike alphaherpesviruses, there are few reports regarding other viral proteins that regulate host shutoff-associated activity in gammaherpesviruses, with the exception of the EBV protein kinase BGLF4 that antagonizes BGLF5-mediated viral gene shutoff [73]. Thus, it is necessary to continue exploring the viral proteins regulate SOX or muSOX activity after gammaherpesvirus infection.

In addition, ICP27 facilitates viral RNA export by recruiting mRNA export adaptors to viral replication sites and binding intronless viral mRNAs through its RGG domain [22, 74–78]. ICP27 promotes expression of the full-length gC protein and tightly regulates the expression of HSV-2 monocistronic ICP34.5 mRNA by inhibiting splicing and activating a cryptic polyadenylation signal (PAS) in new introns [23]. The PRV UL54 protein is a homolog of HSV-1 ICP27 and has a drastic impact of the genome-wide expression of PRV genes, especially on the transcription of the true late genes [79]. These studies have provided insights into the crucial role of ICP27 and its homologs in selectively regulating viral mRNA nuclear export to favor viral RNA transcription and protein translation.

### Immune evasion

The detection of microbial pathogens is an essential first step in mounting an innate immune response to infection. Pattern recognition receptors (PRRs) recognize pathogen-associated molecular patterns (PAMPs) and trigger the production of numerous host defense molecules, including interferons (IFNs), proinflammatory cytokines and chemokines [80]. In addition, IFNs can be classified into three groups (types I, II and III), where IFN-I (IFN-α/β) and III are crucial antiviral factors that stimulate the synthesis of a variety of antiviral effector molecules [81]. A number of innate immune mechanisms are invoked following infection, and herpesviruses in turn takes different measures to neutralize these host responses, with host shutoff proteins playing crucial roles in escaping innate immune.

### VHS as an IFN-α/β resistance factor

VHS has been identified as an IFN-α/β resistance factor that is essential for viral survival. Primary murine embryonic fibroblasts (MEFs) infected with HSV-2 ΔVHS mutants were observed to produce > 50-fold more IFN-a/β than cells infected with wild-type and VHS-rescued viruses. In addition, pretreatment of MEFs with IFN-I inhibited the replication of HSV-2 ΔVHS more than that of wild-type and VHS-rescued viruses, indicating that VHS interferes with activation of the IFN-a/β-induced antiviral response. The authors further examined whether VHS interferes with key mediators of the IFN-a/β response, PKR and RNase L [82]. Furthermore, HSV-1 VHS-defective viruses have been shown to induce increased, physiologically active levels of IFN and increased amounts of ISGs. VHS-defective HSV-1 viruses have increased susceptibility to IFN in cells [44], but not in culture, and the virulence of these viruses is not restored in IFN-a/β/γ R^−^/^−^ mice [83]. The HSV-2 VHS protein is ~ 40-fold more active than that of HSV-1 and has a more crucial role in HSV-2 than its HSV-1 counterpart in promoting resistance to the IFN response and plays an important role in damaging the host defense mechanism. In addition, Bovine herpesvirus 1 (BHV-1) ICP27, as a potent IFN-β antagonist, interferes with the promoter activity of IFN-β1 and IFN-β3 [84].

### Inhibition of cellular PRR-mediated antiviral responses

Cyclic-GMP-AMP (cGAMP) synthase (cGAS), the most recently identified cytosolic DNA sensor, plays an important role in IFN-I responses against DNA viruses, including HSV-1 and KSHV. Interestingly, HSV-1 UL41 degrades cGAS mRNAs via its RNase activity to evade the cGAS/STING-mediated DNA-sensing pathway [85]. Furthermore, ICP27 interacts with the TBK1-STING signalosome in the cytoplasm through its RGG motif to inhibit interferon regulatory factors 3 (IRF3) activation and IFN production through the cGAS-STING pathway in macrophages [86]. In addition, HSV-2 ICP27 also directly associates with IRF3 and inhibits its phosphorylation and nuclear translocation, resulting in the inhibition of IFN-β production [87].

IFI16 was identified as a DNA sensor that also signals through STING-TBK1 to detect viral DNA in both the cytoplasm and nucleus [88]. Interestingly, nuclear IFI16 can assemble inflammasomes during infection by KSHV and HSV-1, leading to the secretion of proinflammatory interleukins [89, 90]. HSV-1 rapidly blocks IFI16-mediated immune responses during infection by catalyzing its degradation, in part via the contribution of ICP0 [90]. However, ICP0 is not necessary or sufficient for the loss of IFI16 in a tumor-derived cell line, and the ICP0-independent loss of IFI16 in HeLa cells is dependent in part on VHS RNase activity [91].

**Fig. 1:**
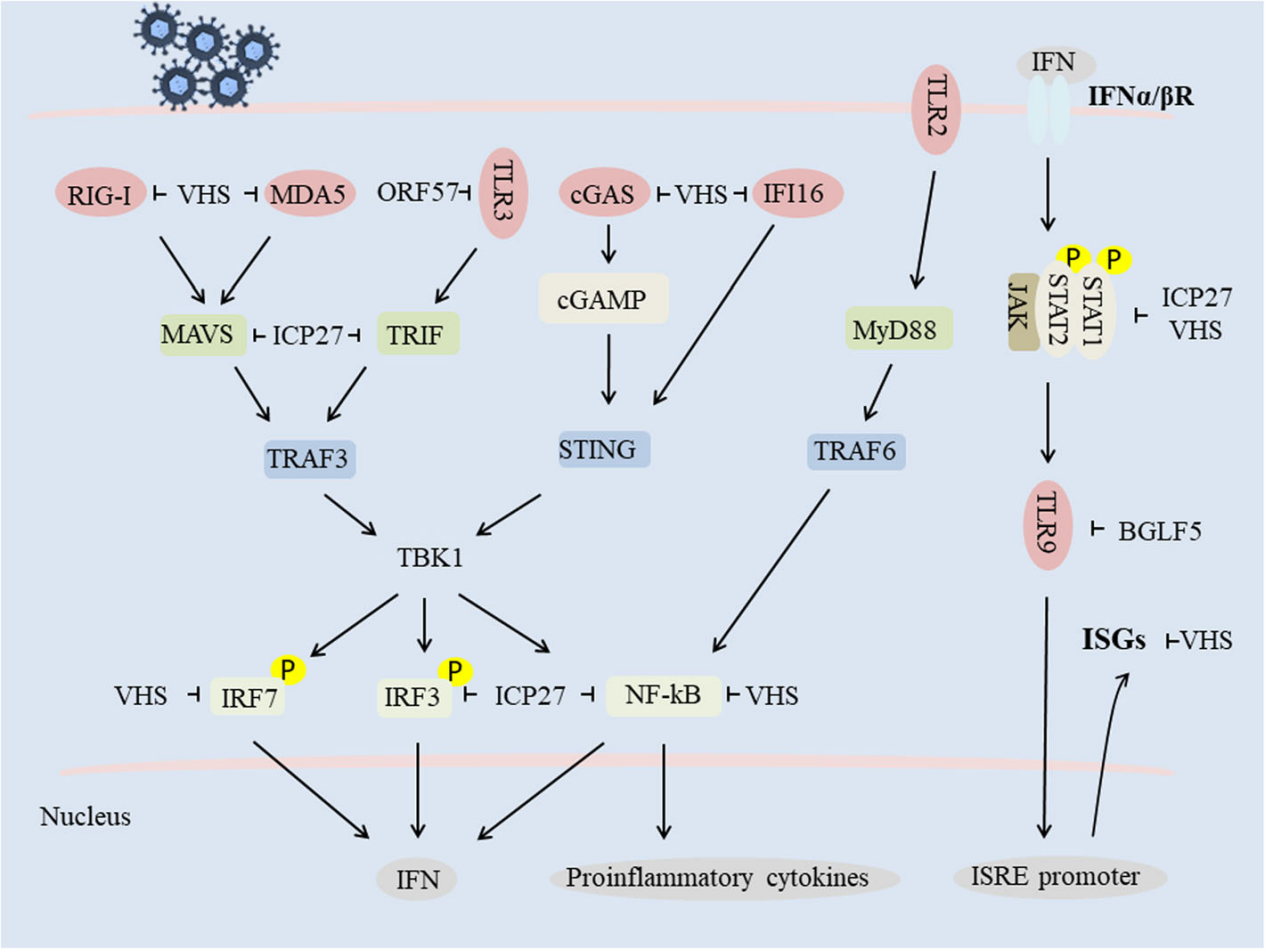
Herpesvirus host shutoff-associated proteins mediate evasion of the type I IFN signaling pathway (refer to [96])

TLRs and RLRs are also fundamental sensor molecules of the host innate immune system that detect conserved molecular signatures of a wide range of microbial pathogens and initiate innate immune responses via distinct signaling pathways [92]. The HSV-2 VHS protein inhibits TLR3 and RIG-I/Mda-5 as well as TLR2-mediated antiviral pathways for sensing dsRNA and effectively suppresses IFN-β antiviral responses in human vaginal epithelial cells (ECs) [93]. ICP27 also inhibits signaling downstream of the RIG-I adaptor protein MAVS and the TLR adaptor protein TRIF, while the KSHV ORF57 protein inhibits TLR3 phosphorylation [45, 94]. In addition, the EBV lytic-phase protein BGLF5 contributes to downregulation of TLR9 levels through RNA degradation [95] (Fig. 1).

### Counteracting ISGs

IFN activates the Janus kinase signal transducer and activator of transcription (JAK/STAT) signaling pathway, resulting in the downstream expression of hundreds of antiviral host effector proteins called ISGs [97, 98]. However, HSV-1 infection restricts the expression of some ISGs through various strategies. For instance, ICP27 downregulates IFN-induced STAT1 phosphorylation and promotes inhibition of STAT1 nuclear accumulation [99]. VHS partially inhibits JAK1 and STAT2 by degrading their mRNAs [100], and the VHS homolog BHV-1 UL41 protein directly binds and cleaves STAT1 mRNA [101]. In addition, VHS degrades some ISG mRNAs through its RNase activity to counteract their antiviral activity, including IFIT3 [102], viperin [103], tetherin [104], ZAP [105], and CH25h [106] (Table 1).

**Table 1:** Inhibition of ISGs

Protein	Pathogen	ISG	Mechanism
VHS	HSV-1	IFIT3	mRNA degradation
		Viperin	
		Tetherin	
		CH25h	
		hZAP	
		PKR	
VHS	PRV	TNF-α	mRNA degradation

### Inhibition of proinflammatory chemokines and cytokines

The VHS protein also suppresses proinflammatory chemokines and cytokines, such as interleukin (IL)-1β, IL-8, macrophage inflammatory protein-1α (MIP1α) [107], and alpha-thalassemia/mental retardation syndrome X-linked (ARTX), an effector of the innate immune response [108], which inhibits major histocompatibility complex (MHC) class I/II and quenches activation of some antigen-presenting dendritic cell (DC) subtypes [93]. Thus, VHS is a crucial determinant of HSV virulence. Similar to VHS, the BGLF5 and SOX proteins downregulate the expression of multiple immune components and reduce the levels of lipid antigen-presenting CD1d and HLA class I /II molecules [109]. However, because this activity is redundant with other EBV proteins that specifically combat HLA processing and transport, it appears to have only a small effect on CD8+ T cell recognition [110, 111]. Selective inactivation of muSOX mRNA degradation activity leads to a severe attenuation of MHV68 in B cells during the phase of peak latency establishment [112]. In addition, ICP27 inhibits p65 acetylation and NF-kB transcriptional activity by repressing Daxx sumoylation [113] (Table 2).

**Table 2:** Host shutoff-associated proteins inhibit various proinflammatory cytokines and cytokines

Protein	Pathogen	Target protein	Mechanism
VHS	HSV-1	IL-1β, IL-8	mRNA degradation
		MIP-1α	
		NF-KB	
		MHC-I/II	
		RNase L	
		JAK1	
		STAT2	
		SOCS3	
VHS	BHV-1	MHC-I/II	mRNA degradation
BGLF5	EBV	HLAI/II	mRNA degradation
		CD1d	
ICP27	HSV-1	NF-KB	Inhibition of NF-kB transcriptional activity by repressing Daxx sumoylation
ICP27	HSV-1	p65	Inhibition of p65 acetylation
ICP27	HSV-1	STAT1	Inhibition of STAT-1 phosphorylation and nuclear accumulation
ICP27	BHV-1	IFNβ	Inhibition of IFN-β1/3 promoter activity

### Suppression of the UPR

Eukaryotic cells respond to various types of stresses caused by changes in the extracellular environment, and the accumulation of unfolded and misfolded proteins in the endoplasmic reticulum (ER) causes ER stress that activates the unfolded protein response (UPR) via three ER transmembrane receptors: PERK, inositol-requiring enzyme 1 (IRE1) and activating transcription factor 6 (ATF6) [114]. The kinase activity of IRE1α leads to activation of c-Jun N-terminal kinases (JNKs) during HSV-1 infection, where ICP27 activates the stress-responsive JNKs to enhance viral replication [115]. VHS suppresses the IRE1/XBP1 signal pathway by directly reducing the accumulation of XBP1 mRNA [116]. Thus, UPR signaling clearly has an important role in immunity and inflammation [117]. The UPR can also support important antiviral responses, activate proinflammatory cytokines and cytokines [118]. Therefore, we speculate that VHS inhibits the UPR pathway to cellular resources for viral replication as well as to promote evasion of the immune response activated by UPR to ensure viral survival. However, unlike VHS, KSHV SOX protein does not affect the expression of UPR genes [119]. These results indicate that different herpesviruses have evolved distinct mechanisms to regulate the UPR to promote viral replication.

### SGs disassembly

**Fig. 2:**
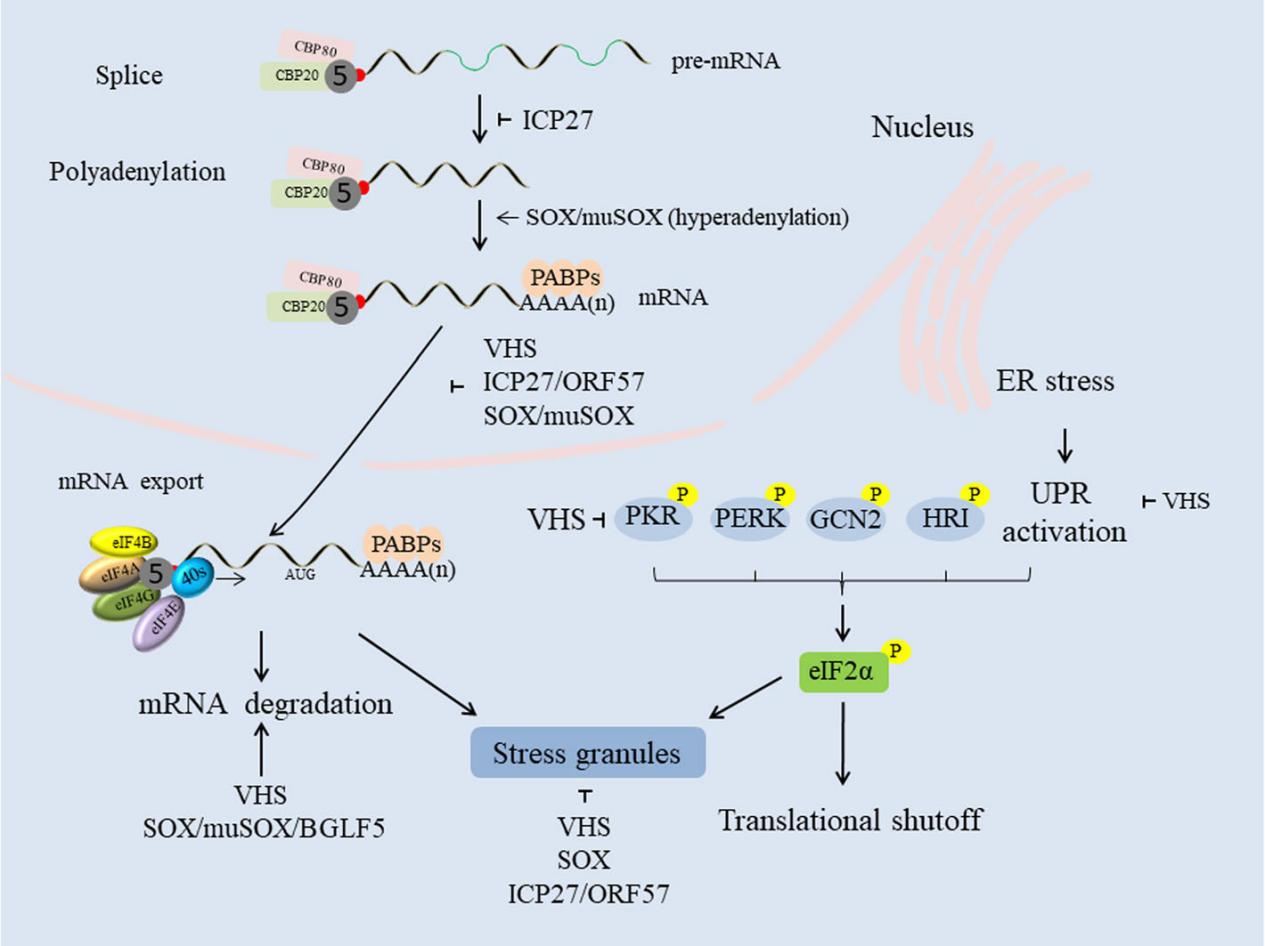
VHS, ICP27 and SOX reduce mRNA abundance to shutoff the expression of host proteins through different strategies. VHS and SOX degrade mRNA via their RNase activity; ICP27 inhibits host pre-mRNA polyadenylation and splicing; and SOX/muSOX proteins induce nascent host mRNA hyperadenylation. In addition, these three proteins alter the localization of cytoplasmic poly (A) binding protein (PABPC), leading to limited mRNA export from the nucleus to the cytoplasm. VHS suppresses the unfolded protein response (UPR) during endoplasmic reticulum (ER) stress, and protein kinase R (PKR) phosphorylates eukaryotic translation initiation factor 2α (eIF2α). VHS and SOX also inhibit the subsequent formation of stress granules (SGs) to favor viral replication (refer to [123])

Stress granule (SG) formation can interfere with viral replication, as herpesviruses require the host translation machinery to synthesize viral proteins. Interestingly, an HSV ΔVHS mutant cannot disrupt arsenite-induced SG formation, an ability that is restored by VHS complementation, and this VHS-mediated disruption also occurs in the absence of other viral proteins [120]. Furthermore, VHS endoribonuclease activity is required to disrupt SG formation, which, in concert with Xrn1 exonuclease activity, promotes the destruction of mRNAs present in existing SGs, leading to their disassembly [121, 122]. Some reports have suggested that SGs act as platforms that sense viral molecular patterns and initiate downstream signaling to promote antiviral responses, as SGs can promote PKR activation in HSV-1 infection, although the ability of VHS to suppress IFN is unrelated to its ability to inhibit PKR activation and SG formation [121]. Furthermore, KSHV SOX also inhibits arsenite-induced SG formation, and HSV-1 ICP27 blocks the PKR/eIF2α/SG pathway to overcome host antiviral responses, an activity that its EBV homolog EB2 lacks [45]. Thus, further exploration of the relationships among of SGs, IFN and ICP27 will be very meaningful (Fig. 2).

## Conclusions

*Herpesviridae* family members are among the most ubiquitous and successful viruses known and are thought to have coevolved with their hosts. The success of herpesviruses is due in part to their use of host shutoff mechanisms to ensure the efficient translation of viral mRNAs while constraining host protein expression. Although accumulating evidence has elucidated these protein-associated host shutoff and immune invasion mechanisms, much remains unclear regarding the biogenesis, characteristics, and adaptive responses of shutoff activity in different viral strains and various hosts. With the exception of HSV, and understanding of the UL41 proteins of other alphaherpesviruses is limited. UL41 proteins from different viruses, such as ORF17 and VHS, have different effects on viral infections. In addition, the HSV-1 VHS protein not only induces mRNA degradation, it also promotes dsRNA degradation. These results suggest that VHS homologs in other alphaherpesviruses may have another function in addition to RNase activity, which should be further explored in future studies. In particular, VHS suppresses a variety of cytokines, which leads to widespread immune shutoff, and whether other host proteins arrest VHS-induced immune shutoff for cell survival is unknown. Furthermore, the fate of AU-rich mRNAs is unknown. AREs generally promote destabilization, and VHS efficiently degrades AU-rich mRNAs by binding TTP and AU-rich mRNAs that are not resistant to SOX-induced shutoff. However, the SRE in the IL-6 mRNA 3’UTR can effectively escape viral endonucleases, and this region also contains AREs. The mechanisms associated with this process are undoubtedly complicated, and the role of viral endonucleases in the fate of AU-rich mRNAs is worth further study. It is unclear whether SOX/muSOX proteins need host or viral proteins to target transcripts. We further speculate that these proteins may play role in other cellular and host processes to promote survival, which should aid in elucidating the mechanisms of herpesviruses host shutoff. In summary, a better understanding of host shutoff proteins not only provides new insights into the viral replication, expression and immune evasion process, but also contributes to provide new molecular targets for the development of antiviral drugs and therapies.

## Data Availability

Not applicable.

## Abbreviations

HSV-1 and HSV-2: Herpes simplex virus types 1 and 2
BHV-1: Bovine herpesvirus 1
HCMV: Human cytomegalovirus
HHV-6: Human Herpesvirus-6
KSHV: Kaposi’s sarcoma-associated herpesvirus
EBV: Epstein-Barr virus
MHV68: murine gammaherpesvirus 68
MDV: Marek’s disease virus
PRV: Pseudorabies virus
VZV: Varicella-zoster virus
VHS: Virion host shutoff
GADD45β: DNA damage-inducible gene 45
TTP: tristetraprolin
SGs: Stress granules
PABPC: Cytoplasmic poly (A) binding protein
PRRs: Pattern recognition receptors
PAMPs: Pathogen-associated molecular patterns
cGAS: Cyclic-GMP-AMP (cGAMP) synthase
IRE1: Inositol-requiring enzyme 1
DC: Dendritic cell
IL: Interleukin
ZAPs: Zinc-finger antiviral proteins
DDX60: DExD/H box helicase
NK: Natural killer

## References

[1] Xie Y, Wu L, Wang M, Cheng A, Yang Q, Wu Y (2019). Alpha-Herpesvirus thymidine kinase genes mediate viral virulence and are potential therapeutic targets. Front Microbiol.

[2] Osterrieder K (2017). Cell biology of herpes Viruse.

[3] Agut H, Bonnafous P, Gautheret-Dejean A (2017). Update on infections with human herpesviruses 6A, 6B, and 7. Med Mal Infect.

[4] Foulon T (1992). Herpesviridae: classification and structure in 1991. Comp Immunol Microbiol Infect Dis.

[5] Suazo PA, Ibanez FJ, Retamal-Diaz AR, Paz-Fiblas MV, Bueno SM, Kalergis AM (2015). Evasion of early antiviral responses by herpes simplex viruses. Mediat Inflamm.

[6] Read GS (2013). Virus-encoded endonucleases: expected and novel functions. Wiley Interdiscip Rev RNA.

[7] Glaunsinger B, Ganem D (2004). Lytic KSHV infection inhibits host gene expression by accelerating global mRNA turnover. Mol Cell.

[8] Covarrubias S, Richner JM, Clyde K, Lee YJ, Glaunsinger BA (2009). Host shutoff is a conserved phenotype of gammaherpesvirus infection and is orchestrated exclusively from the cytoplasm. J Virol.

[9] Narayanan K, Ramirez SI, Lokugamage KG, Makino S (2015). Coronavirus nonstructural protein 1: common and distinct functions in the regulation of host and viral gene expression. Virus Res.

[10] Levene RE, Gaglia MM (2018). Host shutoff in influenza a virus: many means to an end. Viruses..

[11] Rivas HG, Schmaling SK, Gaglia MM (2016). Shutoff of host gene expression in influenza a virus and Herpesviruses: similar mechanisms and common themes. Viruses..

[12] Gaglia MM, Covarrubias S, Wong W, Glaunsinger BA (2012). A common strategy for host RNA degradation by divergent viruses. J Virol.

[13] Hardwicke MA, Sandri-Goldin RM (1994). The herpes simplex virus regulatory protein ICP27 contributes to the decrease in cellular mRNA levels during infection. J Virol.

[14] Patel V, Dahlroth SL, Rajakannan V, Ho HT, Cornvik T, Nordlund P (2015). Structure of the C-terminal domain of the multifunctional ICP27 protein from herpes simplex virus 1. J Virol.

[15] Zhu H, Cong J-P, Mamtora G, Gingeras T, Shenk T (1998). Cellular gene expression altered by human cytomegalovirus: global monitoring with oligonucleotide arrays. Proc Natl Acad Sci.

[16] Smiley JR (2004). Herpes simplex virus Virion host shutoff protein: immune evasion mediated by a viral RNase?. J Virol.

[17] Taddeo B, Roizman B (2006). The virion host shutoff protein (UL41) of herpes simplex virus 1 is an endoribonuclease with a substrate specificity similar to that of RNase a. J Virol.

[18] Lin HW, Hsu WL, Chang YY, Jan MS, Wong ML, Chang TJ (2010). Role of the UL41 protein of pseudorabies virus in host shutoff, pathogenesis and induction of TNF-α expression. J Vet Med Sci.

[19] Desloges N, Rahaus M, Wolff MH (2005). The varicella-zoster virus-mediated delayed host shutoff: open reading frame 17 has no major function, whereas immediate-early 63 protein represses heterologous gene expression. Microbes Infect.

[20] Sato H, Callanan LD, Pesnicak L, Krogmann T, Cohen JI (2002). Varicella-zoster virus (VZV) ORF17 protein induces RNA cleavage and is critical for replication of VZV at 37 degrees C but not 33 degrees C. J Virol.

[21] Dauber B, Saffran HA, Smiley JR (2019). The herpes simplex virus host shutoff (vhs) RNase limits accumulation of double stranded RNA in infected cells: evidence for accelerated decay of duplex RNA. PLoS Pathog.

[22] Sandri-Goldin RM (2011). The many roles of the highly interactive HSV protein ICP27, a key regulator of infection. Future Microbiol.

[23] Tang S, Patel A, Krause PR (2019). Hidden regulation of herpes simplex virus 1 pre-mRNA splicing and polyadenylation by virally encoded immediate early gene ICP27. PLoS Pathog.

[24] Tunnicliffe RB, Hu WK, Wu MY, Levy C, Mould AP, McKenzie EA (2019). Molecular mechanism of SR protein kinase 1 inhibition by the herpes virus protein ICP27. mBio.

[25] Tang S, Patel A, Krause PR (2016). Herpes simplex virus ICP27 regulates alternative pre-mRNA polyadenylation and splicing in a sequence-dependent manner. Proc Natl Acad Sci U S A.

[26] Rutkowski AJ, Erhard F, L'Hernault A, Bonfert T, Schilhabel M, Crump C (2015). Widespread disruption of host transcription termination in HSV-1 infection. Nat Commun.

[27] Wang X, Hennig T, Whisnant AW, Erhard F, Prusty BK, Friedel CC (2020). Herpes simplex virus blocks host transcription termination via the bimodal activities of ICP27. Nat Commun.

[28] Rowe M, Glaunsinger B, Leeuwen DV, Zuo J, Sweetman D, Ganem D (2007). Host shutoff during productive Epstein-Barr virus infection is mediated by BGLF5 and may contribute to immune evasion. Proc Natl Acad Sci U S A.

[29] Gaglia MM, Rycroft CH, Glaunsinger BA (2015). Transcriptome-wide cleavage site mapping on cellular mRNAs reveals features underlying sequence-specific cleavage by the viral Ribonuclease SOX. PLoS Pathog.

[30] Covarrubias S, Gaglia MM, Kumar GR, Wong W, Jackson AO, Glaunsinger BA (2011). Coordinated destruction of cellular messages in translation complexes by the gammaherpesvirus host shutoff factor and the mammalian exonuclease Xrn1. PLoS Pathog.

[31] Glaunsinger B, Chavez L, Ganem D (2005). The exonuclease and host shutoff functions of the SOX protein of Kaposi\s sarcoma-associated Herpesvirus are genetically separable. J Virol.

[32] Lee YJ, Glaunsinger BA (2009). Aberrant herpesvirus-induced polyadenylation correlates with cellular messenger RNA destruction. PLoS Biol.

[33] Abernathy E, Clyde K, Yeasmin R, Krug LT, Burlingame A, Coscoy L (2014). Gammaherpesviral gene expression and virion composition are broadly controlled by accelerated mRNA degradation. PLoS Pathog.

[34] Abernathy E, Glaunsinger B (2015). Emerging roles for RNA degradation in viral replication and antiviral defense. Virology..

[35] Feederle R, Bannert H, Lips H, Muller-Lantzsch N, Delecluse HJ (2009). The Epstein-Barr virus alkaline exonuclease BGLF5 serves pleiotropic functions in virus replication. J Virol.

[36] Bagneris C, Briggs LC, Savva R, Ebrahimi B, Barrett TE (2011). Crystal structure of a KSHV-SOX-DNA complex: insights into the molecular mechanisms underlying DNase activity and host shutoff. Nucleic Acids Res.

[37] Muller M, Hutin S, Marigold O, Li KH, Burlingame A, Glaunsinger BA (2015). A ribonucleoprotein complex protects the interleukin-6 mRNA from degradation by distinct herpesviral endonucleases. PLoS Pathog.

[38] Rodriguez W, Srivastav K, Muller M. C19ORF66 broadly escapes virus-induced endonuclease cleavage and restricts Kaposi's sarcoma-associated Herpesvirus. J Virol. 93:2019, e00373–e02019. 10.1128/JVI.00373-19 PMC6613750 30944177

[39] Muller M, Glaunsinger BA (2017). Nuclease escape elements protect messenger RNA against cleavage by multiple viral endonucleases. PLoS Pathog.

[40] Clyde K, Glaunsinger BA (2011). Deep sequencing reveals direct targets of gammaherpesvirus-induced mRNA decay and suggests that multiple mechanisms govern cellular transcript escape. PLoS One.

[41] Esclatine A, Taddeo B, Evans L, Roizman B (2004). The herpes simplex virus 1 UL41 gene-dependent destabilization of cellular RNAs is selective and may be sequence-specific. Proc Natl Acad Sci U S A.

[42] Onomoto K, Yoneyama M, Fung G, Kato H, Fujita T (2014). Antiviral innate immunity and stress granule responses. Trends Immunol.

[43] Sciortino MT, Parisi T, Siracusano G, Mastino A, Taddeo B, Roizman B (2013). The virion host shutoff RNase plays a key role in blocking the activation of protein kinase R in cells infected with herpes simplex virus 1. J Virol.

[44] Pasieka TJ, Lu B, Crosby SD, Wylie KM, Morrison LA, Alexander DE (2008). Herpes simplex virus virion host shutoff attenuates establishment of the antiviral state. J Virol.

[45] Sharma NR, Majerciak V, Kruhlak MJ, Zheng ZM (2017). KSHV inhibits stress granule formation by viral ORF57 blocking PKR activation. PLoS Pathog.

[46] Feng P, Everly DN, Read GS (2001). mRNA decay during herpesvirus infections: interaction between a putative viral nuclease and a cellular translation factor. J Virol.

[47] Doepker RC, Hsu WL, Saffran HA, Smiley JR (2004). Herpes simplex virus Virion host shutoff protein is stimulated by translation initiation factors eIF4B and eIF4H. J Virol.

[48] Sarma N, Agarwal D, Shiflett LA, Read GS (2008). Small interfering RNAs that deplete the cellular translation factor eIF4H impede mRNA degradation by the virion host shutoff protein of herpes simplex virus. J Virol.

[49] Teo CSH, O'Hare P (2018). A bimodal switch in global protein translation coupled to eIF4H relocalisation during advancing cell-cell transmission of herpes simplex virus. PLoS Pathog.

[50] Page HG, Read GS (2010). The virion host shutoff endonuclease (UL41) of herpes simplex virus interacts with the cellular cap-binding complex eIF4F. J Virol.

[51] Feng P, Everly DN, Read GS (2005). mRNA decay during herpes simplex virus (HSV) infections: protein-protein interactions involving the HSV virion host shutoff protein and translation factors eIF4H and eIF4A. J Virol.

[52] Shu M, Taddeo B, Roizman B (2015). Tristetraprolin recruits the herpes simplex Virion host shutoff RNase to AU-rich elements in stress response mRNAs to enable their cleavage. J Virol.

[53] Saffran HA, Read GS, Smiley JR (2010). Evidence for translational regulation by the herpes simplex virus virion host shutoff protein. J Virol.

[54] Wigington CP, Williams KR, Meers MP, Bassell GJ, Corbett AH (2014). Poly(a) RNA-binding proteins and polyadenosine RNA: new members and novel functions. Wiley Interdiscip Rev RNA..

[55] Dobrikova E, Shveygert M, Walters R, Gromeier M (2010). Herpes simplex virus proteins ICP27 and UL47 associate with polyadenylate-binding protein and control its subcellular distribution. J Virol.

[56] Salaun C, MacDonald AI, Larralde O, Howard L, Lochtie K, Burgess HM, et al. Poly(a)-binding protein 1 partially relocalizes to the nucleus during herpes simplex virus type 1 infection in an ICP27-independent manner and does not inhibit virus replication. J Virol. 84:2010, 8539–8548. 10.1128/JVI.00668-10 PMC2919032 20573819

[57] Kumar GR, Glaunsinger BA (2010). Nuclear import of cytoplasmic poly(a) binding protein restricts gene expression via hyperadenylation and nuclear retention of mRNA. Mol Cell Biol.

[58] Rwp S, Anderson RC, Larralde O (2017). Viral and cellular mRNA-specific activators harness PABP and eIF4G to promote translation initiation downstream of cap binding. Proc Natl Acad Sci U S A.

[59] Massimelli MJ, Majerciak V, Kruhlak M, Zheng ZM (2013). Interplay between polyadenylate-binding protein 1 and Kaposi's sarcoma-associated herpesvirus ORF57 in accumulation of polyadenylated nuclear RNA, a viral long noncoding RNA. J Virol.

[60] Horst D, Burmeister WP, Boer IG, van Leeuwen D, Buisson M, Gorbalenya AE (2012). The "bridge" in the Epstein-Barr virus alkaline exonuclease protein BGLF5 contributes to shutoff activity during productive infection. J Virol.

[61] Dauber B, Pelletier J, Smiley JR (2011). The herpes simplex virus 1 vhs protein enhances translation of viral true late mRNAs and virus production in a cell type-dependent manner. J Virol.

[62] Dauber B, Poon D, Dos Santos T, Duguay BA, Mehta N, Saffran HA (2016). The herpes simplex virus Virion host shutoff protein enhances translation of viral true late mRNAs independently of suppressing protein kinase R and stress granule formation. J Virol.

[63] Taddeo B, Zhang W, Roizman B (2013). The herpes simplex virus host shutoff RNase degrades cellular and viral mRNAs made before infection but not viral mRNA made after infection. J Virol.

[64] Dauber B, Saffran HA, Smiley JR (2014). The herpes simplex virus 1 virion host shutoff protein enhances translation of viral late mRNAs by preventing mRNA overload. J Virol.

[65] Sadek J, Read GS (2016). The splicing history of an mRNA affects its level of translation and sensitivity to cleavage by the Virion host shutoff endonuclease during herpes simplex virus infections. J Virol.

[66] Shu M, Taddeo B, Zhang W, Roizman B (2013). Selective degradation of mRNAs by the HSV host shutoff RNase is regulated by the UL47 tegument protein. Proc Natl Acad Sci U S A.

[67] Elliott G, Pheasant K, Ebert-Keel K, Stylianou J, Franklyn A, Jones J (2018). Multiple posttranscriptional strategies to regulate the herpes simplex virus 1 vhs Endoribonuclease. J Virol.

[68] Pheasant K, Moller-Levet CS, Jones J, Depledge D, Breuer J, Elliott G (2018). Nuclear-cytoplasmic compartmentalization of the herpes simplex virus 1 infected cell transcriptome is co-ordinated by the viral endoribonuclease vhs and cofactors to facilitate the translation of late proteins. PLoS Pathog.

[69] Shu M, Taddeo B, Roizman B (2013). The nuclear-cytoplasmic shuttling of Virion host shutoff RNase is enabled by pUL47 and an embedded nuclear export signal and defines the sites of degradation of AU-rich and stable cellular mRNAs. J Virol.

[70] Taddeo B, Zhang W, Roizman B (2010). Role of herpes simplex virus ICP27 in the degradation of mRNA by virion host shutoff RNase. J Virol.

[71] Soliman TM, Sandri-Goldin RM, Silverstein SJ (1997). Shuttling ofthe herpes simplex virus type 1 regulatory protein ICP27 between the nucleus and cytoplasm mediates the expression of late proteins. J Virol.

[72] Donnelly M, Elliott G (2001). Nuclear localization and shuttling of herpes simplex virus tegument protein VP13/14. J Virol.

[73] Feederle R, Mehl-Lautscham AM, Bannert H, Delecluse HJ (2009). The Epstein-Barr virus protein kinase BGLF4 and the exonuclease BGLF5 have opposite effects on the regulation of viral protein production. J Virol.

[74] Tian X, Devi-Rao G, Golovanov AP, Sandri-Goldin RM (2013). The interaction of the cellular export adaptor protein Aly/REF with ICP27 contributes to the efficiency of herpes simplex virus 1 mRNA export. J Virol.

[75] Ote I, Piette J, Sadzot-Delvaux C (2010). The varicella-zoster virus IE4 protein: a conserved member of the herpesviral mRNA export factors family and a potential alternative target in antiherpetic therapies. Biochem Pharmacol.

[76] Amor S, Strassheim S, Dambrine G, Remy S, Rasschaert D, Laurent S (2011). ICP27 protein of Marek's disease virus interacts with SR proteins and inhibits the splicing of cellular telomerase chTERT and viral vIL8 transcripts. J Gen Virol.

[77] Corbin-Lickfett KA, Rojas S, Li L, Cocco MJ, Sandri-Goldin RM (2010). ICP27 phosphorylation site mutants display altered functional interactions with cellular export factors Aly/REF and TAP/NXF1 but are able to bind herpes simplex virus 1 RNA. J Virol.

[78] Ote I, Lebrun M, Vandevenne P, Bontems S, Medina-Palazon C, Manet E (2009). Varicella-zoster virus IE4 protein interacts with SR proteins and exports mRNAs through the TAP/NXF1 pathway. PLoS One.

[79] Csabai Z, Takacs IF, Snyder M, Boldogkoi Z, Tombacz D (2017). Evaluation of the impact of ul54 gene-deletion on the global transcription and DNA replication of pseudorabies virus. Arch Virol.

[80] Deng L, Zeng Q, Wang M, Cheng A, Jia R, Chen S, Zhu D, Liu M, Yang Q, Wu Y (2018). Suppression of NF-kappaB activity: a viral immune evasion mechanism. Viruses.

[81] Lazear HM, Schoggins JW, Diamond MS (2019). Shared and distinct functions of type I and type III Interferons. Immunity..

[82] Duerst RJ, Morrison LA (2004). Herpes simplex virus 2 virion host shutoff protein interferes with type I interferon production and responsiveness. Virology..

[83] Leib DA, Harrison TE, Laslo KM, Machalek MA, Moorman NJ, Virgin HW (1999). Interferons regulate the phenotype of wild-type and mutant herpes simplex viruses in vivo. J Exp Med.

[84] da Silva LF, Sinani D, Jones C (2012). ICP27 protein encoded by bovine herpesvirus type 1 (bICP27) interferes with promoter activity of the bovine genes encoding beta interferon 1 (IFN-beta1) and IFN-beta3. Virus Res.

[85] Su C, Zheng C (2017). Herpes simplex virus 1 abrogates the cGAS/STING-mediated cytosolic DNA-sensing pathway via its Virion host shutoff protein, UL41. J Virol.

[86] Christensen MH, Jensen SB, Miettinen JJ, Luecke S, Prabakaran T, Reinert LS (2016). HSV-1 ICP27 targets the TBK1-activated STING signalsome to inhibit virus-induced type I IFN expression. EMBO J.

[87] Guan X, Zhang M, Fu M, Luo S, Hu Q (2019). Herpes simplex virus type 2 immediate early protein ICP27 inhibits IFN-beta production in mucosal epithelial cells by antagonizing IRF3 activation. Front Immunol.

[88] Jin T, Perry A, Jiang J, Smith P, Curry JA, Unterholzner L (2012). Structures of the HIN domain: DNA complexes reveal ligand binding and activation mechanisms of the AIM2 inflammasome and IFI16 receptor. Immunity..

[89] Singh VV, Kerur N, Bottero V, Dutta S, Chakraborty S, Ansari MA (2013). Kaposi's sarcoma-associated herpesvirus latency in endothelial and B cells activates gamma interferon-inducible protein 16-mediated inflammasomes. J Virol.

[90] Diner BA, Lum KK, Javitt A, Cristea IM (2015). Interactions of the antiviral factor IFI16 mediate immune signaling and herpes simplex virus-1 immunosuppression. Mol Cell Proteomics.

[91] Orzalli MH, Broekema NM, Knipe DM (2016). Relative contributions of herpes simplex virus 1 ICP0 and vhs to loss of cellular IFI16 vary in different human cell types. J Virol.

[92] Brubaker SW, Bonham KS, Zanoni I, Kagan JC (2015). Innate immune pattern recognition: a cell biological perspective. Annu Rev Immunol.

[93] Yao XD, Rosenthal KL (2011). Herpes simplex virus type 2 virion host shutoff protein suppresses innate dsRNA antiviral pathways in human vaginal epithelial cells. J Gen Virol.

[94] Stempel M, Chan B, Brinkmann MM (2019). Coevolution pays off: Herpesviruses have the license to escape the DNA sensing pathway. Med Microbiol Immunol.

[95] van Gent M, Griffin BD, Berkhoff EG, van Leeuwen D, Boer IG, Buisson M (2011). EBV lytic-phase protein BGLF5 contributes to TLR9 downregulation during productive infection. J Immunol.

[96] Zheng C (2018). Evasion of cytosolic DNA-stimulated innate immune responses by herpes simplex virus 1. J Virol.

[97] Crosse KM, Monson EA, Beard MR, Helbig KJ (2018). Interferon-stimulated genes as enhancers of antiviral innate immune signaling. J Innate Immun.

[98] Iwasaki A (2012). A virological view of innate immune recognition. Annu Rev Microbiol.

[99] Johnson KE, Song B, Knipe DM (2008). Role for herpes simplex virus 1 ICP27 in the inhibition of type I interferon signaling. Virology..

[100] Chee AV, Roizman B (2004). Herpes simplex virus 1 gene products occlude the interferon signaling pathway at multiple sites. J Virol.

[101] Ma W, Wang H, He H (2019). Bovine herpesvirus 1 tegument protein UL41 suppresses antiviral innate immune response via directly targeting STAT1. Vet Microbiol.

[102] Jiang Z, Su C, Zheng C (2016). Herpes simplex virus 1 tegument protein UL41 counteracts IFIT3 antiviral innate immunity. J Virol.

[103] Shen G, Wang K, Wang S, Cai M, Li ML, Zheng C (2014). Herpes simplex virus 1 counteracts viperin via its virion host shutoff protein UL41. J Virol.

[104] Zenner HL, Mauricio R, Banting G, Crump CM (2013). Herpes simplex virus 1 counteracts tetherin restriction via its virion host shutoff activity. J Virol.

[105] Su C, Zhang J, Zheng C (2015). Herpes simplex virus 1 UL41 protein abrogates the antiviral activity of hZAP by degrading its mRNA. Virol J.

[106] You H, Yuan H, Fu W, Su C, Wang W, Cheng T, Zheng C (2017). Herpes simplex virus type 1 abrogates the antiviral activity of Ch25h via its virion host shutoff protein. Antivir Res.

[107] Suzutani T, Nagamine M, Shibaki T, Ogasawara M, Yoshida I, Daikoku T (2000). The role of the UL41 gene of herpes simplex virus type 1 in evasion of non-specific host defence mechanisms during primary infection. J Gen Virol.

[108] Jurak I, Silverstein LB, Sharma M, Coen DM (2012). Herpes simplex virus is equipped with RNA- and protein-based mechanisms to repress expression of ATRX, an effector of intrinsic immunity. J Virol.

[109] van Gent M, Gram AM, Boer IG, Geerdink RJ, Lindenbergh MF (2015). Silencing the shutoff protein of Epstein-Barr virus in productively infected B cells points to (innate) targets for immune evasion. J Gen Virol..

[110] Quinn LL, Zuo J, Abbott RJ, Shannon-Lowe C, Tierney RJ, Hislop AD, Rowe M (2014). Cooperation between Epstein-Barr virus immune evasion proteins spreads protection from CD8+ T cell recognition across all three phases of the lytic cycle. PLoS Pathog.

[111] Jung J, Munz C (2015). Immune control of oncogenic gamma-herpesviruses. Curr Opin Virol.

[112] Richner JM, Clyde K, Pezda AC, Cheng BY, Wang T, Kumar GR (2011). Global mRNA degradation during lytic gammaherpesvirus infection contributes to establishment of viral latency. PLoS Pathog.

[113] Kim JA, Choi MS, Min JS, Kang I, Oh J, Kim JC (2017). HSV-1 ICP27 represses NF-κB activity by regulating Daxx sumoylation. BMB Rep.

[114] Chae HJ, Yoo WH, Lee WS (2015). ER stress and autophagy. Curr Mol Med.

[115] Su A, Wang H, Li Y, Wang X, Chen D, Wu Z. Opposite roles of RNase and kinase activities of inositol-requiring enzyme 1 (IRE1) on HSV-1 replication. Viruses. 2017;9:–E235. 10.3390/v9090235 PMC5618002 28832521

[116] Zhang P, Su C, Jiang Z, Zheng C (2017). Herpes simplex virus 1 UL41 protein suppresses the IRE1/XBP1 signal pathway of the unfolded protein response via its RNase activity. J Virol.

[117] Grootjans J, Kaser A, Kaufman RJ, Blumberg RS (2016). The unfolded protein response in immunity and inflammation. Nat Rev Immunol.

[118] Smith JA (2014). A new paradigm: innate immune sensing of viruses via the unfolded protein response. Front Microbiol.

[119] Johnston BP, Pringle ES, McCormick C (2019). KSHV activates unfolded protein response sensors but suppresses downstream transcriptional responses to support lytic replication. PLoS Pathog.

[120] Finnen RL, Hay TJ, Dauber B, Smiley JR, Banfield BW (2014). The herpes simplex virus 2 virion-associated ribonuclease vhs interferes with stress granule formation. J Virol.

[121] Burgess HM, Mohr I (2018). Defining the role of stress granules in innate immune suppression by the herpes simplex virus 1 Endoribonuclease VHS. J Virol.

[122] Finnen RL, Banfield BW (2016). Alphaherpesvirus subversion of stress-induced translational arrest. Viruses..

[123] Fros JJ, Pijlman GP (2016). Alphavirus infection: host cell shut-off and inhibition of antiviral responses. Viruses..

